# Audience preferences are predicted by temporal reliability of neural processing

**DOI:** 10.1038/ncomms5567

**Published:** 2014-07-29

**Authors:** Jacek P. Dmochowski, Matthew A. Bezdek, Brian P. Abelson, John S. Johnson, Eric H. Schumacher, Lucas C. Parra

**Affiliations:** 1 Department of Biomedical Engineering, City College of New York, 160 Convent Avenue, New York, New York 10027, USA; 2 School of Psychology, Georgia Institute of Technology, 654 Cherry Street, Atlanta, Georgia 30332, USA; 3 Harmony Institute, 54 West 21st Street, New York, New York 10010, USA; 4 Present address: Department of Psychology, Stanford University, 450 Serra Mall, Stanford, California 94305, USA

## Abstract

Naturalistic stimuli evoke highly reliable brain activity across viewers. Here we record neural activity from a group of naive individuals while viewing popular, previously-broadcast television content for which the broad audience response is characterized by social media activity and audience ratings. We find that the level of inter-subject correlation in the evoked encephalographic responses predicts the expressions of interest and preference among thousands. Surprisingly, ratings of the larger audience are predicted with greater accuracy than those of the individuals from whom the neural data is obtained. An additional functional magnetic resonance imaging study employing a separate sample of subjects shows that the level of neural reliability evoked by these stimuli covaries with the amount of blood-oxygenation-level-dependent (BOLD) activation in higher-order visual and auditory regions. Our findings suggest that stimuli which we judge favourably may be those to which our brains respond in a stereotypical manner shared by our peers.

## Abstract (web summary)

Encephalographic brain recordings are often used to characterize neuronal dynamics at the network level in relation to specific behaviours. Here, Dmochowski *et al*. show that neural activity from a few individuals viewing popular media can predict population-level neural activity in thousands of individuals.

## Graphical abstract

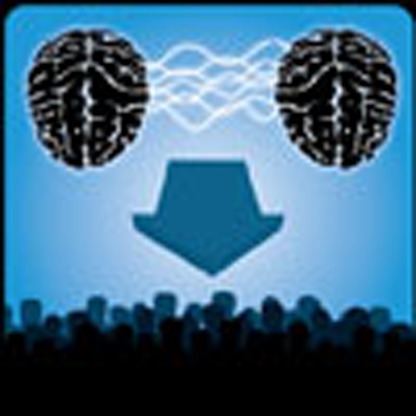

Predicting the behaviour of large groups is inherent to such diverse processes as forecasting election results, anticipating the reception to upcoming films, and foreseeing the effects of changes to laws or policies. Meanwhile, the prediction of individual behaviour is a pillar of neuroscience, with a recent focus on the study of naturally occurring behaviours. Previous investigations have identified the neural correlates of individual preferences^1^,^2^,^3^,^4^,^5^, subjective values^6^ and choices^7^,^8^,^9^ by measuring the functional magnetic resonance imaging (fMRI)-derived blood-oxygenation-level-dependent (BOLD) signal in regions-of-interest while subjects perform experimental tasks. Here we ask whether the neural activity of multiple individuals may collectively predict the behaviour of large groups.

Previous works aimed at predicting population trends from brain activity have employed the amplitude of a neural signal, typically the BOLD, as a readout of future behaviour^4^,^9^,^10^. Such an approach implicitly assumes that the strength of neural response in a fixed region correlates with behavioural measures. More recently, however, a growing link is emerging between the reliability of neural processing (that is, correlation across repeated presentations of the stimulus) and natural behaviours. Indeed, naturalistic audiovisual stimuli have been shown to elicit highly reliable neural activity across multiple viewers^11^, with the level of such inter-subject correlation (ISC) linked to successful memory encoding^12^ and effective communication between individuals^13^. ISC is increased during scenes marked by high arousal and negative emotional valence^11^,^14^, and is strongest for familiar and naturalistic events^15^. In addition to these fMRI studies, recent work found that engaging narrative stimuli yield high levels of ISC in the evoked encephalographic responses of a small sample of viewers^16^,^17^.

Given the evidence linking ISC—inherently a group measure—to brain states characterized by heightened affect, attention and memory retention, we suspected that the agreement in neural responses may serve as a suitable predictor for subsequent population behaviour. Specifically, we hypothesized that the level of neural reliability elicited by a naturalistic stimulus in a small sample would be predictive, to some degree, of behavioural responses reflecting engagement or interest of a large population.

Broadcasts of popular television shows or advertisements serve as a convenient framework for testing our hypothesis: in the social media age, the responses of large audiences are captured in online networks such as Twitter, Facebook and YouTube. We leverage this to explore the link between neural and behavioural responses. Namely, we recruited a sample of 12–16 naive subjects and presented them with stimuli which had been previously aired and for which we compiled aggregated measures of the population response. We imaged brain activity during this exposure, employing electroencephalography (EEG) which captures broad patterns of activity on the time scale of neuronal processing, allowing us to measure reliability in short temporal segments. To further characterize the observed reliability, we subsequently performed an EEG-informed fMRI activation study to identify brain areas which are systematically more (or less) active during stimuli which elicit greater ISCs in the EEG. Most importantly, we found a statistically significant link between the neural reliability in the sample and preferences of large audiences within and across contemporary audiovisual stimuli. Our findings suggest that behavioural responses of large groups to natural stimuli may be robustly predicted from the reliability in corresponding neural responses of a small sample of individuals.

## Results

We sought a stimulus eliciting time-varying and readily available viewer responses across a large population. To that end, we considered the premiere broadcast of a popular television series (‘The Walking Dead’, AMC, 2010) in conjunction with two metrics which capture the audience’s response to the original broadcast in a time-resolved manner.

An online service which collects Twitter traffic information was employed to obtain a comprehensive listing of time-stamped, stimulus-relevant tweets, which originated during the airing of the episode. Meanwhile, 16 study participants representative of the series’ target demographic were recruited to view the episode while having their neural activity recorded with high-density EEG.

**Figure 1:**
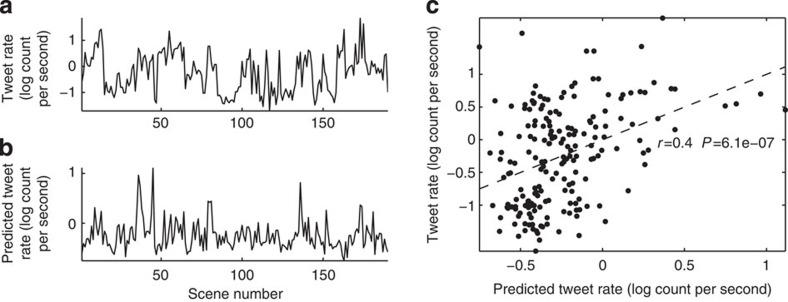
Neural reliability predicts scene-by-scene tweet frequency. (**a**) Log frequency of tweets related to each scene (*N*=190) of a popular television broadcast. (**b**) Log tweet frequency as predicted from the scene-by-scene neural reliability measured across 16 participants during subsequent viewing of the episode in the laboratory (see equation (4) in Methods). (**c**) Neural reliability explains 16% of the variance in the log tweet rate (*r*=0.4, *P*=6.1 × 10^−7^, *N*=190; 95% confidence interval on *r*: (0.26,0.51)). Dashed line represents regression from predicted to actual log tweet rate.

The stimulus was partitioned into its 190 constituent scenes (ranging in duration from 1.4 to 300.5 s, with a median length of 17 s), where a scene was defined as an aggregate of shots (that is, uninterrupted sequences of frames) comprising a distinct narrative event. For each scene, we computed the frequency of elicited tweets. To account for the non-negativity and heavy-tailed distribution of Twitter activity^18^, we logarithmically transformed the tweet rate to yield the time series shown in Fig. 1a, which defines our dependent measure.

Meanwhile, we sought to measure the amount of neural reliability evoked by each scene in our sample of participants. Rather than computing reliability in an electrode-to-electrode fashion, we first performed a dimensionality reduction technique which projects the neural responses from all subjects onto a space which maximizes the ISCs across our sample (see Methods for details of computation). When measured in this optimized space, the bulk of the reliability is captured in just a few dimensions (that is, 3). The resulting scene-by-scene neural reliability was then regressed onto our dependent measure, yielding the predicted log tweet frequency (see equation (4) in Methods) shown in Fig. 1b.

The neural reliability experienced by the sample throughout each scene explains 16% of the variance in audience log tweet frequency (Fig. 1c; *r*=0.40, *P*=6.1 × 10^−7^, *N*=190, *P*-value computed using the analytic distribution of the sample correlation coefficient^19^, 95% confidence interval on *r*: (0.26,0.51) computed using the bootstrap^20^). It is worthwhile to note that while tweeting is a delayed behavioural response, the observed neural reliability is driven by immediate short-term responses (reliability was calculated for activity 0.5 Hz or higher; see Methods and Fig. 4).

**Figure 2:**
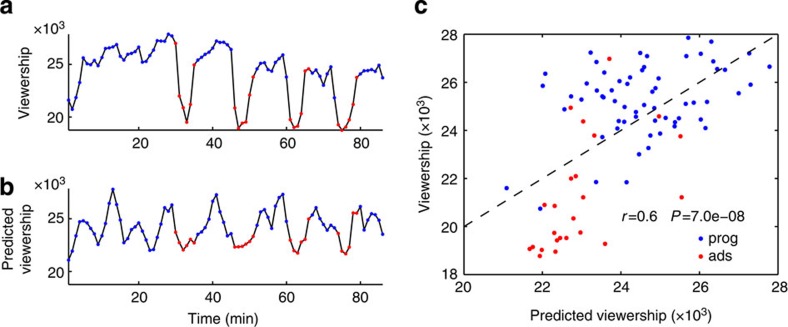
Neural reliability predicts viewership size. (**a**) Viewership size during broadcast of television show as measured by Nielsen ratings. Programming (blue) is interrupted by advertising (red). (**b**) Viewership as predicted from the neural reliability exhibited by 16 participants viewing the same programming. (**c**) Neural reliability explains 36% of the variance in viewership size (*r*=0.60) when including both periods of programming (blue) and advertising (red), while accounting for 34% of the variance during programming alone (*r*=0.58). Dashed line denotes regression from predicted to actual viewership (including ads); prog ads, programming advertisements.

On the basis of previous findings suggesting an association between ISC and narrative quality, novelty and coherence^11^,^16^,^21^, we suspected that neural reliability may also predict viewership size. To that end, we obtained minute-by-minute Nielsen ratings stemming from the original broadcast (including advertisements), resulting in a time series conveying audience size and defining our dependent measure (Fig. 2a).

The decision to continue viewing may depend, in part, on recent viewing history. As a result, we opted not to correlate reliability instantaneously with viewership. Instead, we formed our neural reliability measure using ISCs computed over the prior 3 min of viewing. We then regressed the resulting time series onto the minute-by-minute viewership, yielding the predicted time series shown in Fig. 2b. Neural reliability explains 36% of viewership (Fig. 2c; *r*=0.60, *P*=7.1 × 10^−8^, *N*=86; 95% confidence interval on *r*: (0.45,0.71)).

There are two evident sources of variability in the Nielsen ratings: a sudden drop in ratings during advertisements, and a gradual decay due to declining audience retention. To determine if the measured correlation is driven by the obvious variation from intervening advertisements, we repeated the calculation but omitting the advertising segments. Reliability explains 34% of of the variance during programming alone (*r*=0.58, *P*=2.6 × 10^−5^, *N*=62, 95% confidence interval on *r*: (0.33,0.75)).

The gradual drop in viewership size observed here is typical of the free-viewing environment of the general audience (that is, being able to change the channel at any time). This contrasts with the laboratory environment in which participants are asked to view the entire episode. To compensate for this mismatch in viewing conditions, we removed the linear trend in the viewership size and found even stronger correlations (complete broadcast: *r*=0.68, *P*=4.9 × 10^−11^, 95% confidence interval on *r*: (0.56,0.77); programming only: *r*=0.66, *P*=3.1 × 10^−7^, 95% confidence interval on *r*: (0.45,0.82)). In other words, neural reliability explains 43% of the variance in viewership size during programming after accounting for the drop in retention.

We also considered the effect of the temporal window size (that is, 3 min) used to define reliability on the prediction accuracy. As shown in Supplementary Fig. 1, the strength of the relationship between neural reliability and viewership exhibits a broad peak at a window size of 3–4 min when predicting ratings during both programming and advertisements, while increasing monotonically from 1 to 6 min when excluding ads (see also Supplementary Note 1). In addition, the correlation of viewership size with neural reliability is insensitive to which of the two age categories provided by Nielsen is being predicted (Supplementary Table 1 and Supplementary Note 2).

**Figure 3:**
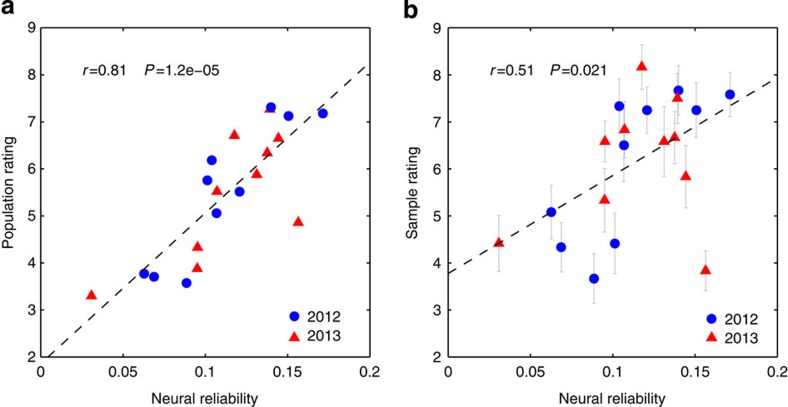
Neural reliability in small sample is predictive of preference ratings in large audience. (**a**) Vertical axis: subjective ratings for 2 × 10 SuperBowl advertisements collected from a large online audience (Facebook-USA Today Ad Meter). Horizontal axis: neural reliability experienced across 12 subjects during each 30 s advertisement. Dashed line indicates the linear prediction of population ratings from neural reliability: 66% of variance in population ratings is explained. (**b**) Same as **a**, but with the vertical axis showing the mean ratings of the individuals in the sample (error bars indicate s.e.m., *N*=12). Only 26% of variance in the sample ratings is explained (*P*=0.047, *N*=20, Fisher *r*-to-*z* transformation).

Both the tweet frequencies and Nielsen ratings considered above quantify audience response during a single programme. Audience preferences are often expressed not within but across competing programming. We wanted to test the ability of the sample neural reliability to predict across-stimuli preferences. We thus obtained the results of a popular online survey occurring annually, in which a large number of participants view and subsequently rate a series of advertisements (SuperBowl commercials). We randomly sampled 10 ads from the 2012 version of this survey and recruited a new set of *N*=12 volunteers to view these ads while recording their EEG. Subjects also provided their own preference rating following the recording. For each advertisement, we computed the neural reliability from the ISCs in the neural responses of the sample (see Methods for details). We found a strong and statistically significant correlation between neural reliability and the population ratings (Fig. 3a, circled markers; *r*=0.90, *P*=9 × 10^−5^, *N*=10, 95% confidence interval on *r*: (0.76,0.97)). Given this surprisingly strong correlation, we sought to validate the results on a new stimulus set, repeating the experiment with the 2013 series of ads while employing the same 12 participants. The neural reliability correlated significantly with the population ratings (Fig. 3a, triangle markers; *r*=0.73, *P*=0.014, *N*=10; 95% confidence interval on *r*: (−0.06,0.95); the drop in correlation from 2012 is driven by a single advertisement, see Supplementary Note 3). By combining all 20 advertisements viewed by each study participant, neural reliability explains 66% of the variance in population ratings (Fig. 3a; *r*=0.81, *P*=3 × 10^−6^, *N*=20, 95% confidence interval on *r*: (0.50,0.92)). Intriguingly, neural reliability explains just 26% of the sample’s own preferences (Fig. 3b; *r*=0.51, *P*=0.02, *N*=20, 95% confidence interval on *r*: (−0.14,0.78)), which is significantly lower than the predictability of the population preferences (*P*=0.047, *N*=20, Fisher *r*-to-*z* transformation).

Could the reduced predictability of the sample ratings result from the variability due to the smaller sample size? To examine this, we generated *N*=10^6^ random samples of 12 ratings for each of the 20 ads (assuming normal distributed ratings with the population rating as the mean and variance as observed in the actual sample). The resulting correlation of these simulated sample ratings with the neural reliability was significantly higher than what was observed for the actual sample ratings: a mean of *r*=0.75 with a 95% confidence interval of (0.66,0.83), leading to a probability *P*=4 × 10^−5^ of drawing the actual value of *r*=0.51 from this distribution. We also explored the possibility of a systematic difference in the ratings of the sample and those of the population. However, ratings were largely consistent, differing significantly for only two of the 20 ads (*P*>0.05 false-discovery rate, *N*=12, Student *t*-test). A positive bias observed in the average rating (+0.65, *P*=0.006, *N*=20, Student *t*-test) should not affect correlation coefficients which are insensitive to a mean offset. Indeed, our sample ratings explain 59% of the variance in the population ratings (*r*=0.77, Supplementary Fig. 2), further lending credence to the notion that the reliability in neural responses is indeed more strongly linked to preferences of the population. Finally, it is worth noting that stimuli were judged with high preference heterogeneity: the same advertising was judged very differently by different subjects (the range of ratings for each ad was 6.25±0.97, that is, almost the full range of 1–10 was used by the 12 subjects to rate the ads).

To probe the spatial dimension of the observed neural reliability, we performed a follow-up fMRI experiment using a separate sample (*N*=14) of individuals, recording the BOLD signal evoked by all 20 of the SuperBowl ads. The subsequent BOLD activation time series were regressed onto the neural reliability scores (see horizontal axis of Fig. 3) in a block-design fashion. We sought to identify brain regions which exhibit systematically higher levels of activation for stimuli marked by high levels of neural reliability.

**Figure 4:**
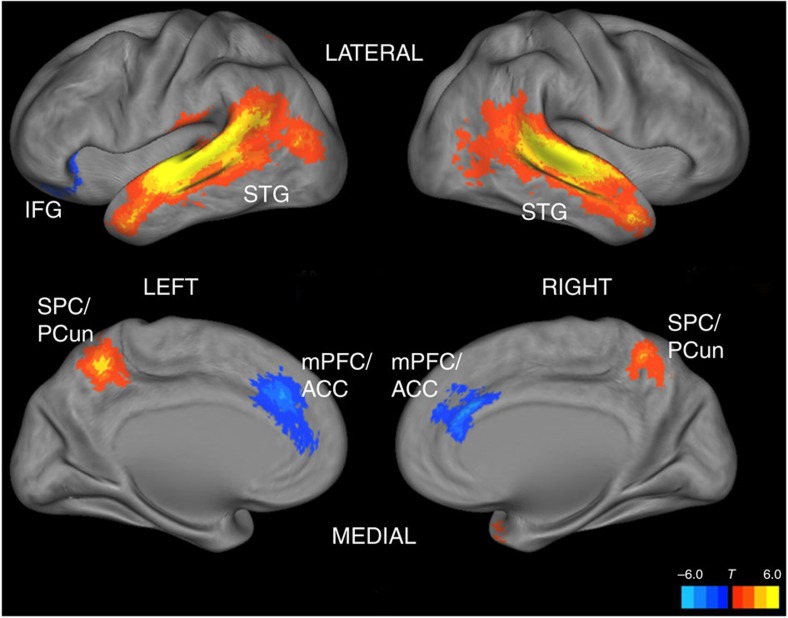
Covariation of BOLD activity with EEG-derived neural reliability in different brain regions. Significant clusters of activation with a corrected family-wise error rate of 0.05 are mapped on inflated cortices using the CARET software^54^. IFG, inferior frontal gyrus; STG, superior temporal gyrus; SPC/PCun, superior parietal cortex/precuneus; mPFC/ACC, medial prefrontal cortex/anterior cingulate cortex. Note that the BOLD and EEG data were collected from separate groups of subjects.

We found significant covariation of BOLD activity with EEG-derived neural reliability in both left and right lateral temporal cortices: these large clusters stretched from sensory association areas in occipital cortex, along the superior temporal gyrus, to the temporal poles (Fig. 4). Moreover, we observed significantly larger BOLD activation patterns for high-reliability advertisements in an area of parietal cortex including the superior parietal lobule and precuneus. Meanwhile, a significant negative covariation between neural reliability and BOLD activation was found in a region of medial prefrontal cortex (mPFC) that includes anterior cingulate cortex (ACC), as well as the left inferior frontal gyrus (IFG). To test if reliability of BOLD activity is also predictive of preference ratings, we computed the ISC of the spatiotemporal patterns of BOLD activity in the identified regions for each advertisement. The measured BOLD-ISC did not significantly correlate with the population nor the sample ratings (*r*=0.34 and *r*=0.23, respectively, *P*>0.14).

## Discussion

Here, we showed that measures of behavioural responses aggregated over large audiences correlate significantly with the neural reliability evoked by the corresponding naturalistic stimuli in a small group of individuals. In particular, neural reliability is highly predictive of across-stimuli preferences, and predicts preferences of the large audience more accurately than those of the individuals from whom the neural activity was recorded.

Our finding differs subtly but importantly from those in which population responses are better predicted from a sample’s neural activity than from its self-reports^4^,^9^. Such findings may, in part, be explained by the fact that the behaviours of the population and sample are being evaluated with somewhat different measures (for example, expressing a preference for a stimulus versus actually consuming it). In the SuperBowl experiment described here, the behaviours performed by both the sample and population are identical, and their responses are well correlated. Note, however, that it is the population ratings that link most strongly to the reliability of neural responses, even though the sample is the source of the measured reliability. We have not found a precedent for the present observation that neural signals explain the population response better than the response of the sample.

One may conclude from the results that stimuli which evoke highly reliable neural responses among a small sample also do so in a larger audience. However, this interpretation does not account for the finding of significantly lower predictability of the sample ratings, which cannot be fully explained from the reduction in sample size. We conjecture that this finding is related to the preference heterogeneity of the advertising stimuli used: the high variability observed in the sample ratings may be attributed to differing subjective values^2^,^22^,^23^ or other variables such as social conformity^24^,^25^. Such idiosyncratic processes may involve complex reasoning or emotional considerations that take relatively long to evaluate and presumably fail to yield immediate and reliable EEG signals. Through population aggregation, however, these idiosyncratic preferences tend to average out and one is left with what is shared by the large audience. Therefore, the surprising finding of this study is that reliability of relatively fast neural processing is a genuine predictor of the common preferences of a large population.

Preference heterogeneity has been studied extensively in the context of economic risk-taking, often focusing on the neural underpinnings of individual differences in decision-making^26^. In the marketing literature, preference heterogeneity has been reported to affect perception of advertising^27^,^28^. However, we are not aware of literature analysing the neural basis of preference heterogeneity with natural stimuli or, in particular, video advertisements.

Traditional neuroimaging work on the evaluation of preference or ‘value’ uses fMRI and points to elevated activity in specific subcortical regions^29^. In particular, activity in the ventral striatum and medial prefrontal cortex (mPFC) correlates with individual subjective value^22^ and the purchasing behaviours of a larger population^4^. Such neural activity encodes information that is predictive of decisions following stimulus presentation^7^,^30^ even when measured in the absence of a choice^31^, thus pointing to a certain level of automatic stimulus evaluation. The present findings highlight the importance of reliable short-latency responses, suggesting similarly automatic stimulus processing. However, the present study points to neural processing of more superficial cortical areas, which are the main contributors to the EEG^32^. Note also that it is the reliability of temporal dynamics, and not necessarily the strength of response, that is carrying the predictive information here. It is also worthwhile to point out that previous efforts at analysing reliability of electrophysiological signals required subdural electrodes and focused on slow modulations (in the order of 10 s) of oscillatory activity, in particular, the gamma band^33^, which is known to correlate with the BOLD response^34^. In contrast, here we used fast evoked responses measured on the scalp, which generally do not coincide with BOLD or gamma activity^35^.

We observed that reliability of neural experience is related to subsequent preferences. However, note that in the Twitter and Nielsen studies, our behavioural measures index general response independent of valence; strictly speaking, the Tweet rates and Nielsen ratings are not reflective of ‘liking’ the stimulus, but rather being compelled to respond to or continue viewing it, respectively. Although it may be argued that tweeting about or tuning into a programme are behaviours consistent with ‘liking’ it, they are certainly not sufficient conditions for doing so. The present analysis has implicitly grouped both positive and negative valences into the dependent measure being predicted: for example, Twitter commentary to the episode expressed both positive as well as negative sentiment. It is thus possible that reliability correlates more generally with, for example, interest, rather than preference itself. On an anecdotal level, we do point out that the SuperBowl ad receiving the lowest population rating (unambiguously denoting a dislike) among all 20 ads also elicited the lowest neural reliability (see Supplementary Table 2).

It is interesting to contrast the present results with the literature on the neural basis of individual differences^36^,^37^,^38^. There, the focus is on capturing neural features which vary across individuals and thus explain differences in individual behaviour. Here, we focus on the commonality in neural responses, effectively ignoring individual differences, to obtain a predictor of group behaviour. The component analysis technique used here to compute neural reliability explicitly looks for shared neural components, and the resulting quantity links closely to population measures which reflect shared behaviours (that is, trends) that emerge after aggregation of large samples.

**Figure 5:**
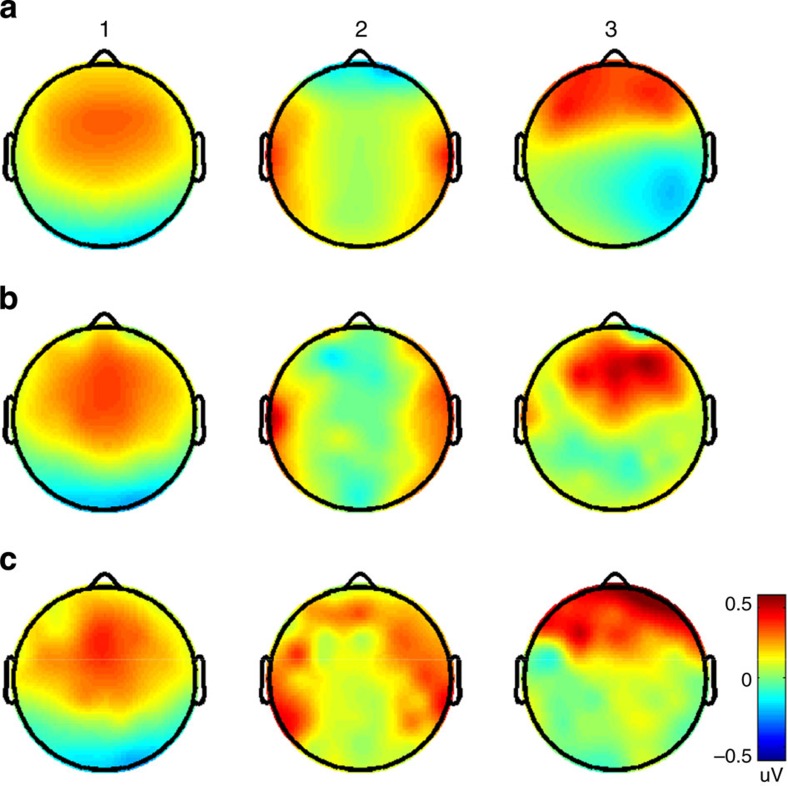
Scalp projections of the three most-reliable dimensions of neural activity. (**a**) As measured during viewing of ‘The Walking Dead’ pilot, (**b**) as measured during viewing of 10 advertisements from the 2012 SuperBowl (**c**) same as **b** but from the 2013 SuperBowl.

The broad fMRI activations observed in sensory and association cortex suggest that modulations of high-level visual and auditory processing underlie the measured neural reliability. For example, the activations in bilateral temporal cortex may reflect processing of complex auditory speech information (both linguistic and prosodic) during advertisements^39^. The activated region also included areas of occipitotemporal cortex recruited by the processing of dynamic visual stimuli^40^,^41^. Increased BOLD activation was also found in the superior parietal lobules and precuneus, which mediates attention to auditory and visual stimuli^42^,^43^,^44^. In addition, this region has also been associated with self-referential processing, imagery and memory^45^, processes that may be elicited during the viewing of well-crafted advertising. Meanwhile, activations of the ACC/mPFC have been implicated in the evaluation of conflict and emotions^46^, which may have occurred more frequently during the less likable advertisements. We caution, however, that all of these observed BOLD activations were found to co-vary with the EEG reliability of a separate group of subjects; as such, we refrain from inferring that the encephalographic signal components driving our preference-linked measure of neural reliability originate from these fMRI-identified regions. Although the topographies of the EEG components have been found to be fairly reproducible across various stimuli (see Fig. 5), the specific co-varying BOLD activations may be stimulus-dependent. Disparate neuromodulatory processes may manifest in similar patterns of cortical generators which drive the observable EEG^32^.

It is possible that personal preferences yield changes in the individual’s level of attention or engagement. Such ‘top-down’ modulation may then affect the strength^47^ and thus the reliability of neural responses associated with stimulus-locked neural processing. However, if individual preferences were to guide modulation of sensory processing, then we would have expected neural reliability to predict the sample preferences equally well, if not better, than the population preferences. Alternatively, it may be that individuals prefer stimuli precisely because the narratives drive the brain strongly and reliably. Such ‘bottom-up’ influence would evidently be well reflected in the preferences of large audiences; however, in the small sample, this sensory processing may be masked by the idiosyncratic preferences or biases of particular individuals.

The findings of the SuperBowl advertisement study suggest that stimuli which we judge favourably may be those to which our brains respond in a stereotypical manner that is shared by our peers. Viewed in another manner, if one is able to evoke reliable neural activity from one’s audience, then that audience is, as a whole, more likely to find one’s message favourable. However, the present data do not permit causal inference about the specific variables mediating the reliable patterns of activity. One possibility is that narrative elements of the stimuli directly bring about neural reliability. Indeed, disrupting the narrative structure for stimuli is known to reduce ISC for BOLD^48^ and evoked responses^16^. But it is also possible that other aspects of the stimulus (for example, overall production quality) correlate with population preference^49^, with this hidden variable explaining the link between advertisement ratings neural reliability. In this case, if one were to pinpoint the stimulus features that drive neural reliability, it would be possible to make the prediction of population behaviour directly from a content analysis of the stimulus (that is, without measuring neural responses).

Regardless of the source of the reliability-preference link, the finding that naturally occurring audience behaviours may be forecast from scalp measurements bears potentially tremendous relevance for fields outside the basic sciences such as education, marketing and media, which stand to gain from the predictive power of neural reliability.

## Methods

### Subjects and stimuli

For the encephalography recordings, 16 healthy subjects (nine females and seven males, ages 19–32, mean of 26 years) viewed the pilot episode of ‘The Walking Dead’ along with intermittent commercials as aired in the original broadcast. An additional 12 subjects (gender balanced, ages 20–29, mean of 25 years) viewed and subsequently rated (on a scale of 1–10) 10 advertisements initially aired during the 2012 SuperBowl (one subject was common to both experiments). To validate the results, the same subjects then viewed 10 ads from the 2013 SuperBowl. These 20 video clips were randomly selected and spanned the range of viewer ratings from the Facebook-USA Today Ad Meter (see Supplementary Table 2). For the fMRI recordings, a separate 14 subjects (six females, ages 18–22, mean of 20 years) viewed the same set of 2012 and 2013 SuperBowl advertisements. Subjects provided written informed consent in accordance with the procedures approved by the Institutional Review Boards of the City College of New York (EEG study) and the Georgia Institute of Technology (fMRI study).

### EEG data collection

Study participants viewed the stimuli in a darkened, electrically and acoustically shielded room. Sound was played back with PC loudspeakers adjusted by each subject to a comfortable listening volume. Subjects were instructed to pay attention to the stimuli and to minimize overt movement. Before viewing, subjects were fitted with a 64-electrode cap placed on the scalp according to the international 10/10 standard for EEG, which was recorded with a BioSemi ActiveTwo system (BioSemi, Amsterdam, The Netherlands) at a sampling frequency of 512 Hz and 24 bits per sample. To subsequently correct eye-movement artifacts, we also recorded the electrooculogram (EOG) with four auxiliary electrodes (one adjacent to and one below each eye).

### EEG preprocessing

All data processing was performed automatically (that is, with no manual intervention) offline in the MATLAB software (MathWorks, Natick, MA, USA). After extracting the EEG/EOG segments corresponding to the duration of each stimulus, the signals were high-pass filtered (1 Hz cutoff), notch filtered at 60 Hz, and down sampled to 256 Hz. Eye-movement related artifacts were corrected by linearly regressing out the four EOG channels from all EEG channels. The regression was performed on non-overlapping 5-s blocks for The Walking Dead data set, and on the entire data record for each SuperBowl advertisement (that is, a 30-s ‘window’). After the correction of eye-movement artifacts, channels whose average power exceeded the mean channel power by four standard deviations were excluded from analysis, with this process repeated four times in an iterative scheme. Similarly, within each kept channel, samples whose squared-amplitude exceeded the mean-squared-amplitude of that channel by more than four standard deviations were rejected. Again, this procedure was iterated four times for each channel. In addition, we rejected every sample within 100 ms of the identified artifactual samples. As our viewing paradigm did not constrain the subjects’ eye movements during the relatively long stimulus durations, the data contained a larger proportion of artifacts than that seen in conventional, short-trial-based experiments. The proportion of data rejected for each scene of the Walking Dead episode is shown in Supplementary Fig. 3: there is no significant correlation between the time series and the log tweets per scene (*r*=0.04, *P*>0.05), nor between the time series and the prediction of log tweets per scene from neural reliability (*r*=0.002, *P*>0.05). Meanwhile, the proportion of data rejected for each SuperBowl ad is listed in Supplementary Table 2: there is no significant correlation between the proportion of data removed for each ad and the prediction of rating from the ISC (*r*=0.24, *P*>0.05), nor between the proportion of data removed for each ad and the population rating (*r*=0.21, *P*>0.05), nor between the proportion of data removed and the sample rating (*r*=0.09, *P*>0.05). In summary, the median (across subjects) percentage of samples removed was 15.98% for the Walking Dead data set, 16.28% for the 2012 SuperBowl data set and 19.95% for the 2013 SuperBowl data set. Rejected samples were marked as missing data (‘NaN’), and the analysis proceeded by computing means and covariances with the *nanmean()* and *nancov()* MATLAB functions. As detailed in the next section, the method employed to compute reliability is rooted in covariance matrices whose sensitivity to outliers is well known; thus, we opted for a stringent outlier rejection to ensure robust covariance estimation.

### Neural reliability computation

To compute the neural reliability elicited by a given stimulus, we employed the component analysis approach of Dmochowski *et al*.^16^, whose mathematical details are described below. The technique is similar to canonical correlation analysis^50^ and its generalizations to multiple subjects^51^, differing in that it uses the same projection for all data sets. It is conceptually similar to the ‘common canonical covariates’ method^52^, which is based on a maximum-likelihood formulation, as opposed to the generalized eigenvalue problem developed in ref. 16.

For a given stimulus viewed by *N* subjects, we have a set of *N* data matrices {**X**_1_,…, **X**_*N*_} where **X**_*n*_ conveys the spatiotemporal neural response of subject *n*. We seek to project the data of all subjects onto a common space such that the resulting projections exhibit maximal ISCs across the subject pool. To that end, let *p*_*i*_ = {*p*_*i*1_, *p*_*i*2_} = {(1,2),(1,3),…,(*N*−1,*N*)} denote the set of all *P*=*N* × (*N*−1)/2 unique subject pairs. We then form the aggregated auto- and cross-covariance matrices as:


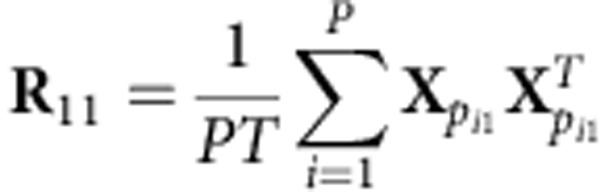



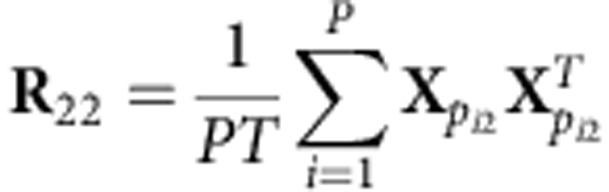



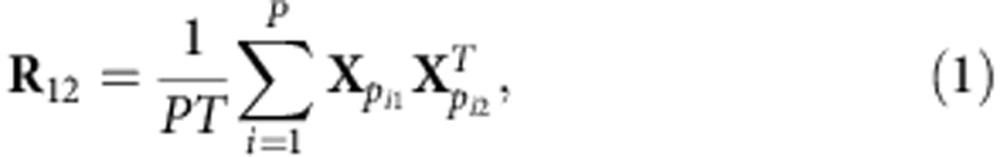


where *T* is the number of time samples (columns) in **X**_*n*_ and ^*T*^ denotes matrix transposition.

We seek to find a projection vector **w** which maximizes the ISC between subject-aggregated data records:


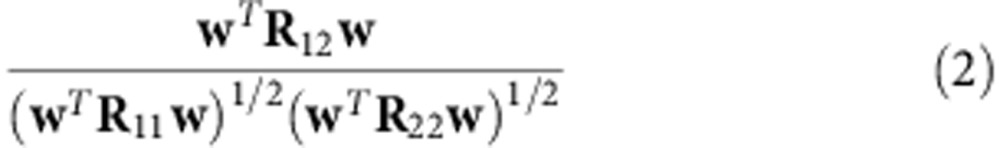


It is shown in ref. 16 that assuming **w**^*T*^**R**_11_**w** = **w**^*T*^**R**_22_**w**, the solution to equation (2) is a generalized eigenvalue problem:


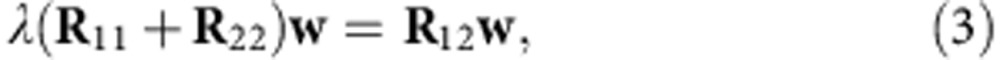


where *λ* is the generalized eigenvalue corresponding the maximal ISC, encompassing all subject pairs, elicited by the stimulus. Note that the assumption **w**^*T*^**R**_11_**w** = **w**^*T*^**R**_22_**w** does not limit generality, as one can simply define *p*_*i*_′={(1, 2),…, (*N*−1, *N*), (*N*, *N*−1),…, (2, 1)} and then substitute *p*_*i*_′ in equation (1) to ensure that **R**_11_=**R**_22_; this was done in our analysis. Moreover, when computing the generalized eigenvalues of equation (3), we regularize the pooled auto-covariance by keeping only the first *K*=10 dimensions. This value of *K* roughly corresponds to the ‘knee’ of the pooled auto-covariance eigenvalue spectrum in the spectral representation of **R**_11_+**R**_22_.

There are multiple non-orthogonal solutions to equation (3), whose associated generalized eigenvalues are ranked in decreasing order of aggregated ISC: *λ*_1_ > *λ*_2_ > … > *λ*_*D*_, where *D* is the number of electrodes. We take the first *C*=3 such solutions and linearly sum their corresponding eigenvalues to yield the estimate of the population measure:


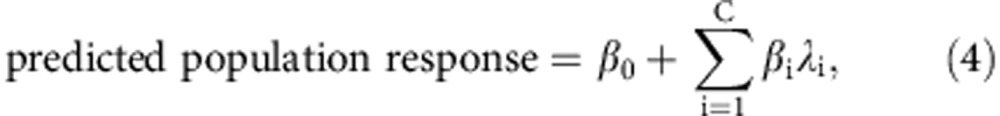


where *β*_*i*_ is the regression coefficient relating the aggregated ISC in dimension *i* to the dependent population measure, as determined by linear least-squares. Note that due to the small sample size of the SuperBowl data set, the ISCs there were uniformly summed across components to yield the estimate of neural reliability which was then directly correlated with the population measure:


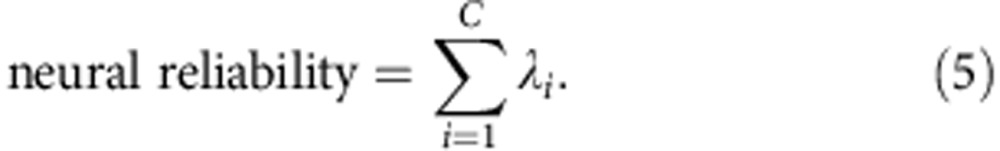


For The Walking Dead data set, we learned the optimal projections on data encompassing all scenes, and then applied these projections back onto the data of each scene to yield the time-resolved reliability in each component. In other words, the covariances in equation (1) were formed using data from all scenes, yielding the optimal **w**, which was then applied to equation (2) but with the covariances there formed using only data for the desired segment of the stimulus. For the SuperBowl data set, we learned the optimal projections by concatenating the neural responses of all ads into a single data matrix per subject. Once again, this combined data was used to construct the covariance matrices and learn the optimal projection vectors. We then projected these optimized filters onto the data from each advertisement to compute the reliability exhibited by the participants’ during that ad.

### Spatiotemporal characteristics of EEG components

Here we detail the spatial and temporal properties of the components formed from the optimal spatial filters **w**. Let us construct a weight matrix **W** whose columns represent the first *C* generalized eigenvectors in equation (3). The projections of the resulting components onto the scalp data are given by Parra *et al*.^53^:


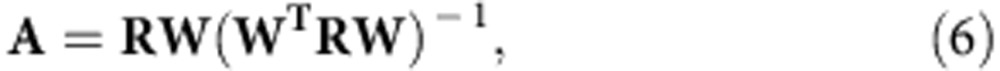


where **R**=**R**_11_+**R**_22_ is the pooled auto-covariance. The columns of **A** are termed ‘forward models’ and inform us of the approximate location of the underlying neuronal sources (up to the inherent limits imposed by volume conduction in EEG).

Figure 5 depicts these forward models for the stimuli used in the study. The scalp projections stemming from The Walking Dead study bear a close resemblance to those found in ref. 16: a symmetric first component with a dipolar distribution consisting of frontocentral and occipital poles, a second component exhibiting bilateral poles at the temporal electrodes, and an asymmetric third component marked by frontal and right-parietal poles. Meanwhile, the forward models of the reliability-maximizing components from the SuperBowl study reveal a highly congruent first component topography, while deviating somewhat in the second and third components. For example, the frontal pole of the third component from the 2012 ads is slightly more posterior. Such disparities in scalp topographies may reflect a re-distribution of canonical sources among the three components.

**Figure 6:**
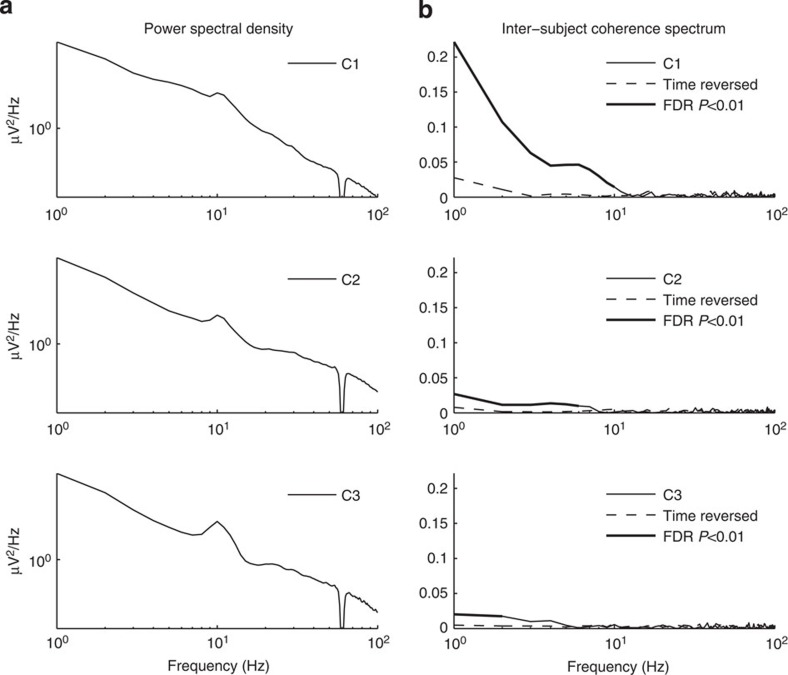
Temporal properties of the signal components used to measure reliability. (**a**) Power-spectral density of each dimension of reliability averaged across all subjects from the 2012 SuperBowl data. A characteristic peak is evident for the alpha band (around 10 Hz). (**b**) Coherence spectrum computed for signals from pairs of subjects (66 unique pairs) and averaged across 10 video clips and all pairs (solid curve). Coherence is a frequency-resolved positive measure of correlation (on a scale of 0–1). Chance level of coherence was estimated by using the same signal pairs with one of the two reversed in time for the entire 30 s of each video clip (dashed curve). Significant difference was calculated using a bootstrap shuffle by pooling original and time-reversed coherence values and randomly drawing (10^5^ times) among the pairs of signals. Significant coherence controlling for false-discovery rate at 0.01 indicated in bold.

Meanwhile, Fig. 6 summarizes the temporal properties of the components used to construct the measure of neural reliability. The 1/f temporal power spectrum of these components is typical for encephalography (Fig. 6a). A temporal coherence analysis of the signals used to measure neural reliability indicates that reliability is driven by immediate evoked responses of 2 s or less and can be as fast as as 100 ms (Fig. 6b). Coherence across subjects—a frequency-resolved measure of correlation—is strongest at low frequencies, but statistically significant values can be found at frequencies as high as 10 Hz, consistent with previous findings using intra-cortical recordings^33^.

### fMRI data collection

For the fMRI recordings, we used the same two sets of ads from the 2012 and 2013 SuperBowls. All MRI data were acquired on a Siemens Magnetom Trio 3T scanner. A high-resolution T1 structural scan (3D MPRAGE, TI=850 ms, flip angle=9°, 1 mm isotropic resolution) was acquired before each subject viewed the ads. Before functional scanning, subjects were instructed to pay attention to the stimuli. Images were acquired using a whole-brain echo-planar imaging sequence (transverse orientation, TR=2,000 ms, TE=30 ms, flip angle=90°, field of view=204 mm) of 37 interleaved slices with 3 mm isotropic resolution and a 17% gap. Data were preprocessed to correct for slice timing to the first slice with a Fourier interpolation, using AFNI’s *3dTshift* tool. Head movements were then corrected using AFNI’s *3dvolreg* routine. Next, the functional data were smoothed with a 6 mm full-width half-maximum Gaussian kernel to reduce noise. Finally, data were transformed to the MNI standard space using FSL’s FLIRT software using a 12-parameter trilinear affine transformation. The EEG reliability measure (see equation (5)) for each advertisement was used as an amplitude-modulated block-design regressor in a general linear model of the fMRI data including six motion parameters as covariates, using AFNI’s *3dDeconvolve* tool. Whole-brain group level analysis was performed using AFNI’s *3dttest* routine, with mixed effects inference on a one-sample *t*-test using individual beta values. AFNI’s *3dClustSim* tool was used with an estimated smoothing of 9.16 mm (obtained with AFNI’s *3dFWHMx* routine) to perform 10 000 Monte Carlo simulations to find a cluster size threshold (40 voxels) with a corrected family-wise error rate of 0.05; *P*(uncorrected)=0.002. To compute the BOLD-ISC for each advertisement, we concatenated the BOLD time series of all significant voxels shown in Fig. 4, and then computed the correlation coefficient between all subject pairs. The resulting aggregated ISCs were then correlated with the population ratings.

### Twitter data collection

Through the Crimson Hexagon service, we obtained a listing of all episode-related tweets which originated during the initial broadcast of The Walking Dead pilot, that is, all tweets from 10/31/2010 9:00–10:00 PM EST containing a relevant hashtag, referencing a show-specific Twitter account, or simply referencing the show’s name. The listing was filtered to retain only those tweets which directly referenced episode content (that is, 1,947 of 19,000 total tweets). Each retained tweet was then manually linked to the corresponding scene(s) by inspecting the message content as well as the tweet timestamp. This procedure was performed by three research assistants who were blind to the hypothesis of the study. Approximately 61% of tweets could be unambiguously linked to one scene only; when ambiguous, the tweet was linked to multiple candidate scenes (that is, in general, the mapping from tweet to scene was one-to-many). The distribution of number of scenes referenced per tweet is shown in Supplementary Fig. 4. The tweet count of each scene was computed by summing the number of tweets referencing that scene. To subsequently analyse the relationship between neural reliability and Twitter reaction, we divided the tweet counts (incremented by one to handle forthcoming log operation) by scene duration to compensate for varying scene length, yielding a tweet-frequency. Finally, we logarithmically transformed the tweet rate to arrive at the dependent measure onto which neural reliability was regressed.

### Nielsen data collection

Courtesy of AMC, we obtained the Nielsen ratings for each minute of the initial airing of pilot episode of The Walking Dead. We summed the ratings across age categories (18–49 and 25–54) and method of viewing (live versus digital video recorder). The regression results when predicting the viewership within each age category are reported in Supplementary Table 1.

### Ad Meter data collection

We obtained publicly available population-averaged scores for all 2012 and 2013 SuperBowl advertisements via the Facebook-USA Today Ad Meter service (Supplementary Table 2). An online panel of over 7,000 participants rated each advertisement on a scale of 1–5 for the 2012 commercials and on a scale of 1–10 for the 2013 commercials. We analysed each set separately and then combined the results. For the combined analysis, the ratings of the 2012 commercials were linearly transformed under the assumption that the quality was comparable to that of 2013: the 2012 ratings were scaled and offset such that the entire set of ratings from 2012 matched the ratings from 2013 in mean and standard deviation.

## Author contributions

J.P.D. and L.C.P. designed EEG experiments, analysed the data and wrote the paper. M.A.B., E.H.S. and L.C.P. designed the fMRI experiment and wrote the corresponding portion of the paper. J.P.D. collected and processed the EEG data. MAB collected and analysed the fMRI data. B.P.A. compiled the Twitter data, proposed the use of and compiled the Nielsen data. J.S.J. facilitated access to the Twitter and Nielsen data sets.

## Additional information

**How to cite this article:** Dmochowski, J. P. *et al*. Audience preferences are predicted by temporal reliability of neural processing. *Nat. Commun.* 5:4567 doi: 10.1038/ncomms5567 (2014).

## Supplementary Material

Supplementary Information Supplementary Figures 1-4, Supplementary Tables 1-2 and Supplementary Notes 1-3
