# Supplementary material for: Audience preferences are predicted by temporal reliability of neural processing
Source: Nat Commun. 2014 Jul 29;5:4567. doi: 10.1038/ncomms5567 (PMC4124862; doi:10.1038/ncomms5567)
Supplement: Supplementary Information — Supplementary Figures 1-4, Supplementary Tables 1-2 and Supplementary Notes 1-3 [file ncomms5567-s1.pdf]

Supplementary material: Audience preferences are predicted by temporal reliability of neural processing

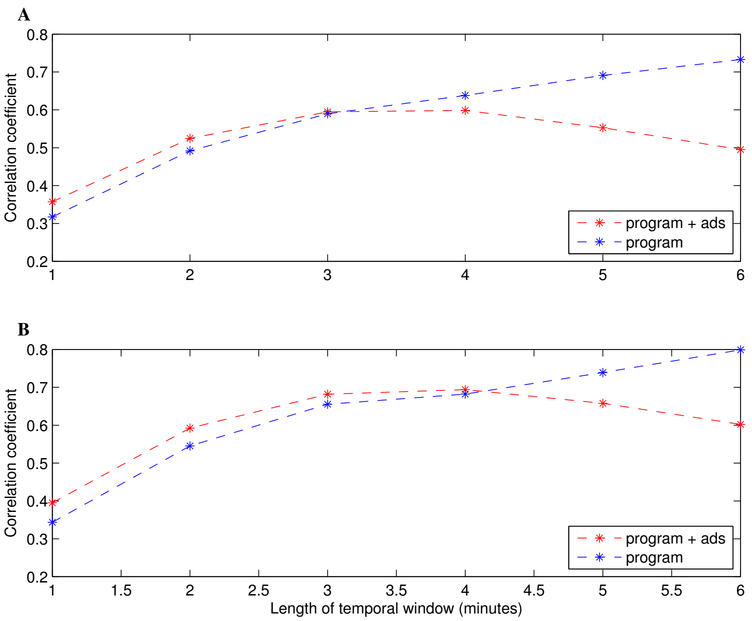

Figure 1: **Effect of temporal window size on relationship of ISC with viewership.** A: When including the ads, peak correlation of ISC with viewership is attained for a window size of 3-4 minutes. Meanwhile, when considering programming only, the correlation of ISC with Nielsen ratings monotonically increases with temporal window size on the range 1-6 minutes. B: same as A, but computed after removing the downward trend in the viewership.

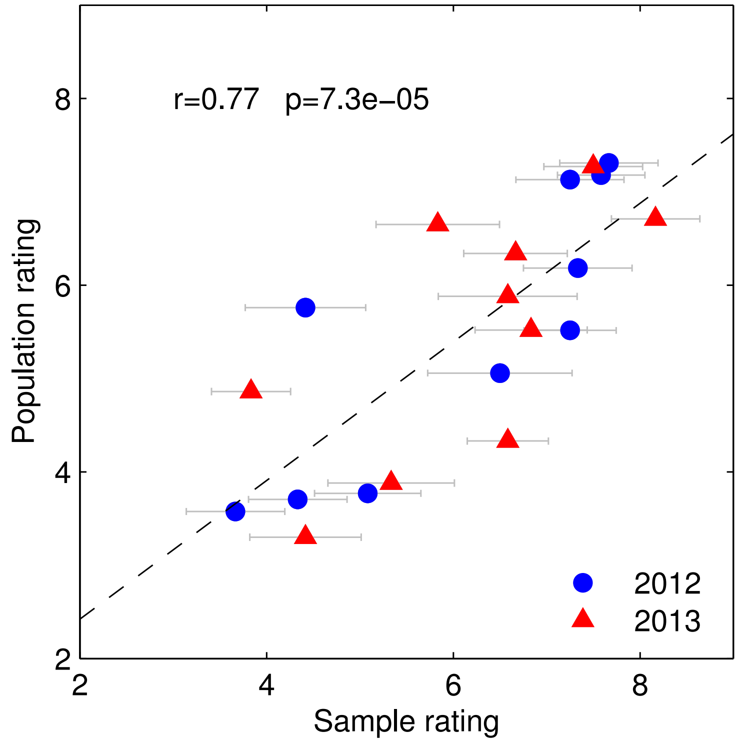

Figure 2: **Population ratings are strongly correlated with those of the sample.** Horizontal axis denotes the mean ratings assigned to each advertisement by the $N = 12$ study participants, with error bars denoting the SEM across subjects. Vertical axis denotes the aggregated ratings of > 7000 online voters. The sample ratings explain 59% of the variance in the population ratings.

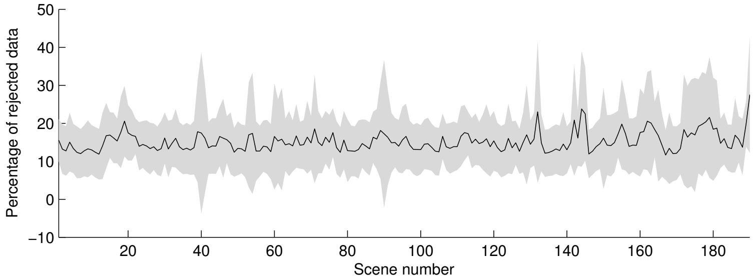

Figure 3: **Percentage of rejected data per scene in the Walking Dead analysis.** Error bars denote the standard error of the mean (SEM) and are computed across the subject dimension ($n = 16$).

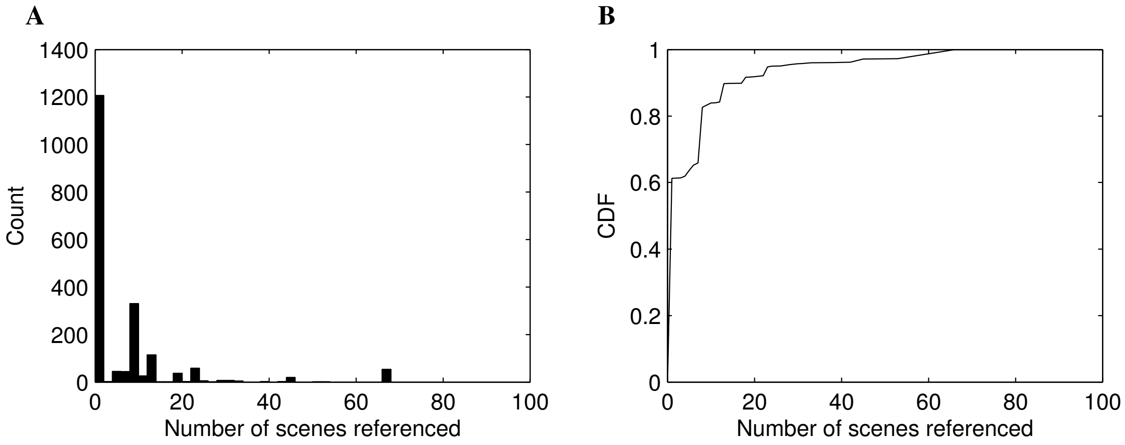

Figure 4: **Number of scenes referenced by each tweet during *The Walking Dead* pilot.** (**A**) The majority of tweets could be unambiguously linked to a single scene: median=1 scene per tweet. When ambiguous, the tweet count of all candidate scenes was incremented, resulting in a mean of 6.88 scenes per tweet. (**B**) The corresponding cumulative density function (CDF).

Table 1: Prediction accuracy for different age categories on the *Nielsen* data set.

Condition	r	p	95% conf. int.
**18-49** (program + ads)	0.63	1.0×10^{-10}	{0.49,0.73}
**18-49** (program only)	0.61	1.4×10^{-7}	{0.40,0.78 }
**18-49** (detrended, program + ads)	0.67	1.2×10^{-12}	{0.54,0..76}
**18-49** (detrended, program only)	0.63	3.2×10^{-8}	{0.41,0.81}
**25-54** (program + ads)	0.58	3.6×10^{-9}	{0.41,0.70}
**25-54** (program only)	0.54	6.3×10^{-6}	{0.32,0.73}
**25-54** (detrended, program + ads)	0.70	6.0×10^{-13}	{0.57,0.77}
**25-54** (detrended, program only)	0.64	1.5×10^{-8}	{0.42,0.82}

Table 2: Neural reliability and sample/population ratings for 2012 and 2013 SuperBowl advertisements.

**Description** (Advertiser)	**Ad Meter** (Rating/10)	**Sample** (Rating/10) mean±std. dev.	**Neural Reliability** (Corr. Coeff.) mean±std. dev.	**Proportion rejected** (%) mean±std. dev.
"Weego" (Bud Light)	7.18*	7.58 ± 1.62	0.17 ± 0.096	19.9 ± 16.8
"Work" (Bud Light Platinum)	3.71*	4.33 ± 1.83	0.069 ± 0.068	17.8 ± 13.8
"Return of the King" (Budweiser)	5.76*	4.42 ± 2.23	0.10 ± 0.069	21.4 ± 16.8
"Sling Baby" (Doritos)	7.31*	7.67 ± 1.83	0.14 ± 0.10	22.1 ± 15.8
"Seduction" (Fiat)	6.18*	7.33 ± 2.02	0.10 ± 0.075	26.3 ± 23.2
"Cloud" (Go Daddy)	3.57*	3.67 ± 1.83	0.089 ± 0.082	20.7 ± 16.1
"Think Fast" (Hyundai)	5.52*	7.25 ± 1.71	0.12 ± 0.073	16.7 ± 10.0
"Superbowl 2012" (Lexus GS)	3.77*	5.08 ± 1.98	0.063 ± 0.080	23.6 ± 16.9
"Just My Shell" (M&M's)	7.13*	7.25 ± 2.01	0.15 ± 0.089	18.3 ± 17.3
"Adriana Lima" (Teleflora)	5.06*	6.50 ± 2.68	0.11 ± 0.054	17.6 ± 14.2
"Lifeguard" (Axe)	4.33	6.58 ± 1.50	0.095 ± 0.091	28.8 ± 28.1
"Puppy is a wolf" (cars.com)	5.52	6.83 ± 2.08	0.11 ± 0.082	24.0 ± 21.7
"Guy in underwear" (Calvin Klein)	3.88	5.33 ± 2.35	0.095 ± 0.077	18.0 ± 11.0
"Fashionista Dad" (Doritos)	7.27	7.50 ± 1.83	0.14 ± 0.090	29.8± 29.6
"Goat 4 Sale" (Doritos)	6.71	8.17 ± 1.64	0.12 ± 0.078	16.7 ± 7.7
"Bar Refaeli make out" (Go Daddy)	3.30	4.42 ± 2.07	0.031 ± 0.076	14.2 ± 5.9
"Kid assembles team" (Hyundai)	6.65	5.83 ± 2.29	0.14 ± 0.089	24.7 ± 23.2
"Love Ballad" (M&M's)	6.34	6.67 ± 1.92	0.14 ± 0.085	22.2 ± 21.5
"Whispering in the library" (Oreo)	5.88	6.58 ± 2.57	0.13 ± 0.088	15.9 ± 7.3
"Jocks love Jared" (Subway)	4.86	3.83 ± 1.47	0.16 ± 0.13	25.0 ± 26.1

*scaled and offset to match mean and standard deviation of 2013 ratings

Supplementary Note 1

Effect of temporal window size on prediction of Nielsen ratings. To predict viewership from neural reliability, we chose a temporal window size of 3 minutes as this value seemed to us to reflect a reasonable time over which to average neural activity to arrive at an estimate of behavior. Figure 1 displays the effect of varying the window size (from 1 to 6 minutes) on the resulting correlation coefficient between predicted and actual viewership. For all window sizes and all conditions (with and without ads, with and without downward trend in viewership), bootstrapped 95% confidence intervals exclude $r = 0$. Moreover, for all window sizes and all conditions, correlation coefficients are statistically significant ($p < 0.01$). When including ads, a broad peak is attained at a window size of 3-4 minutes, while for programming only the prediction accuracy monotonically increases across the selected range. The largest jump in correlation coefficient occurs from 1 to 2 minutes, possibly reflecting the fact that viewership at time t is related to neural reliability on the interval $(t - \tau, t)$, with $\tau > 0$. Hence, matching the temporal window instantaneously to the window used to measure viewership is suboptimal. Note that for a window size of w minutes, the first $w - 1$ minutes lack the sufficient temporal aperture to compute our measure of reliability. To handle this, we simply assumed that the ISC at $t < 0$ was equal to that experienced during the first minute of viewing. Another way of saying this is that for the first $w - 1$ minutes of viewing, we only averaged over the available data.

Supplementary Note 2

Effect of age category on prediction of Nielsen *ratings.* There were two age categories provided by *Nielsen*: 18-49, and 25-54. Our subject pool (ages ranged from 19-32) overlapped with both categories. As a result, we summed the viewership across the two categories to form the viewership time series used in the regression. Here we report the results when considering the categories separately. Table 1 lists the correlation coefficients, p-values, and bootstrapped 95% confidence intervals on r for each age category, where we have also included the results for programming only as well as those obtained after detrending the viewership as described in the manuscript.

Supplementary Note 3

Facebook-USA Today Ad Meter *scores.* The 2012 Ad Meter population scores were obtained from: `http://usatoday30.usatoday.com/money/advertising/story/2012-02-07/usa-today-facebook-super-bowl-ad-meter-final-scores/53004026/1`. The 2013 Ad Meter population scores were obtained from: `http://admeter.usatoday.com/articles/view/the-results`.

For the combined analysis, the ratings of the 2012 commercials were scaled under the assumption that the quality was comparable to that of 2013. The 2012 ratings were scaled and offset such that the entire set of ratings from 2012 ($N = 56$) matched the ratings from 2013 ($N = 54$) in mean and standard deviation (5.61 ± 1.07) using the following: 2012 rescaled ratings = 2012 ratings $\times$ 1.6306 + 0.2483.

The drop in prediction performance of population ratings from neural reliability from 2012 to 2013 is driven by the *Subway* advertisement (2012: $r = 0.90$, 2013: $r = 0.73$). After exclusion, the correlation for 2013 increases to $r = 0.88, p = 0.0018$. This ad is distinctive in that it has repeated and jarring scene cuts throughout its 30 second duration, which may be the source of the relatively strong neural reliability (i.e., stronger than expected given the linear relationship with population rating).
